# ﻿Revision of the genus *Labidolanguria* Fowler, 1908 (Coleoptera, Erotylidae, Languriinae), with descriptions of a new species and two new combinations

**DOI:** 10.3897/zookeys.1233.132046

**Published:** 2025-04-01

**Authors:** Zheng-Zhong Huang, Xing-Ke Yang, Si-Qin Ge

**Affiliations:** 1 Institute of Zoology, Chinese Academy of Sciences, Beijing, China Institute of Zoology, Chinese Academy of Sciences Beijing China; 2 University of Chinese Academy of Sciences, Beijing, China University of Chinese Academy of Sciences Beijing China

**Keywords:** China, Cucujoidea, identification key, lizard beetle, new record, taxonomy

## Abstract

The oriental genus *Labidolanguria* Fowler, 1908, previously remained unknown in China, is revised in this study. A new species, *Labidolangurialiangi* Huang, **sp. nov.**, is described and illustrated from the Xizang Autonomous Region. Two new combinations are proposed: *Labidolanguriaapicata* (Zia, 1959), **comb. nov.**, and *L.sauteri* (Fowler, 1913), **comb. nov.** This genus now comprises four recognized species in Asian, and a key is provided for their identification. The relationship of the genus *Labidolanguria* to closely related genera is discussed.

## ﻿Introduction

The Genus *Labidolanguria* has been monotypic since Fowler (1908) established it in the subfamily Languriinae. In the present study, a new species was discovered and is described here as *Labidolangurialiangi***sp. nov.** Additionally, two species formerly described in another genus are transferred into the genus *Labidolanguria*: *Tetraphalasauteri* (Fowler, 1913) and *Tetraphalaapicata* (Zia, 1959). The number of the species of *Labidolanguria* is increased from one to four, which are mostly distributed in the Himalayan region and southwestern China. An identification key for all known *Labidolanguria* species is also provided.

## ﻿Materials and methods

The material studied is deposited in the following collections:

**IZAS**Institute of Zoology, Chinese Academy of Sciences, Beijing, China

**MNHN**Muséum national d’Histoire naturelle, Paris, France

**NHMUK**Natural History Museum, London, United Kingdom

**SDEI**Senckenberg Deutsches Entomologisches Institut, Müncheberg, Germany

Specimens used in this study were relaxed in distilled water for 12–24 h prior to dissection of the genitalia and mouthparts. Detached parts were soaked in 10% KOH solution for 12–24 h at room temperature, rinsed with distilled water, and dissected in 75% ethanol under a Nikon SMZ1000 stereomicroscope. All photographs of adults were taken by a Canon 5D Mark III digital camera equipped with a Canon MP-E 65 mm macro lens. The images were stacked with Helicon Focus v. 6.7.1 and edited with Adobe Photoshop CS6 to correct contrast, brightness, and imperfections. We utilized a Zeiss Axio Zoom.V16 Fluorescence Stereo Zoom Microscope equipped with an AxioCam MRc 5 camera to acquire images of the male and female genitalia. Subsequently, we conducted photomontage in Zen 2012 (blue edition) imaging software.

The holotype and paratypes of *L.liangi* sp. nov. and *L.apicata* (Zia, 1959) comb. nov. are deposited in IZAS. The syntype of *L.sauteri* (Fowler, 1913) comb. nov. is deposited in SDEI. The holotype of *Labidolanguriamucronata* Fowler, 1908 is deposited in NHMUK.

## ﻿Taxonomy

### ﻿Genus Labidolanguria

Taxon classificationAnimaliaColeopteraErotylidae

Fowler, 1908

B72F7D87-BAB2-5592-A88B-D2B3D672FAAA

Labidolanguria Fowler, 1908: 9. Type species: Labidolanguriamucronata Fowler, 1908 by monotypy.

#### Diagnosis.

Antennal club composed of 4–6 antennomeres. Dorsal body surface with green or deep -green metallic luster. Compound eyes large and finely faceted. Pronotum with basal pronotal carina. Elytral epipleura absent, apex of elytra acute, with outer angle of elytra somewhat acute and produced, sutural angle acute but not produced. Prosternal process rectangular, with two long grooves along each side.

#### Distribution.

China (Xizang, Yunnan, Sichuan, Taiwan), India (Nilgiri Hills).

#### Remarks.

When Fowler (1908) established this genus, he only compared it with the genus *Pentelanguria* Crotch, 1876. *Labidolanguria* and *Pentelanguria* share a similar elytral apex, which is acutely pointed. Apart from the smaller and more slender antennae in *Labidolanguria*, the two genera can also be distinguished by the prosternal process, which is smooth and flat in *Pentelanguria*, whereas in *Labidolanguria* it is grooved laterally.

The genera *Labidolanguria* and *Tetraphala* exhibit strong similarities in the structure of the antennae, pronotum, and prosternal process. However, they can be differentiated by the elytral apex, which is consistently truncate and bears small denticles in *Tetraphala*, while in *Labidolanguria* it is acute and lacks denticles.

##### ﻿Key to species of genus *Labidolanguria*

**Table:** 

1	Club composed of the last four antennomeres (Fig. 3A, B)	**2**
–	Club composed of the last five or six antennomeres	**3**
2	Body with bright metallic green; outer angle of elytra acute (Fig. 1A	***L.mucronata* Fowler, 1908**
–	Body deep copper green, with little metallic luster; outer angle of elytra not acute, sometimes with several denticles (Figs 1D, 2A–D)	***L.liangi* sp. nov.**
3	Club composed of the last five antennomeres; abdomen without any black spots (Fig. 1B)	***L.sauteri* (Fowler, 1913), comb. nov.**
–	Club composed of the last six antennomeres; abdomen with black or green metallic spots (Fig. 1C)	***L.apicata* (Zia, 1959), comb. nov.**

### ﻿ Labidolanguria mucronata

Taxon classificationAnimaliaColeopteraErotylidae

Fowler, 1908

34ECD528-D966-5FEA-B034-E8A4F62D1952

Fig. 1A

Labidolanguria mucronata Fowler, 1908: 9. Type locality: India, Nilgiri Hills. Type depository: NHMUK.

#### Type material examined.

***Holotype***: Type [round label] // Type [red rectangular label] // Nilgiri Hills//Andrewes/Bequest. / B. M. 1922-221. // 623 [in red] // *Labidolanguriamucronata* Fowler/ TYPE [handwriting]//QR code NHMUK 010800985

**Figure 1.**
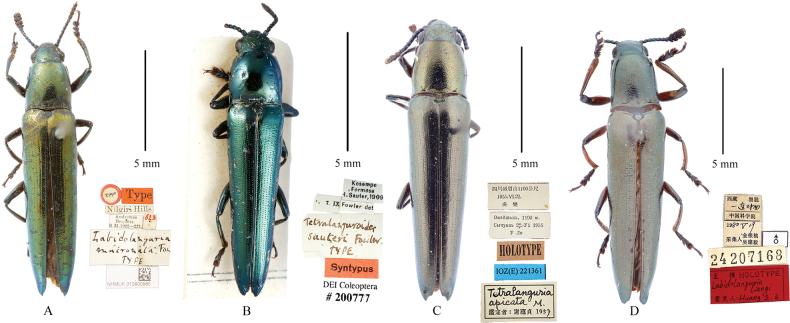
Type materials of all species of *Labidolanguria* with labels pinned under each specimen **A***L.mucronata* Fowler, 1908, holotype **B***L.sauteri* (Fowler, 1913), comb. nov., syntype **C***L.apicata* (Zia, 1959), comb. nov., holotype **D***L.liangi* sp. nov., holotype. Scale bars: 5.0 mm.

#### Additional material examined.

WALLARDI(Travancore) // MUSEUM PARIS/ Ex. Coll. M. MAINDRON/ Coll. G. BABAULT 1930 // *Labidolanguriamucronata* Fowl. / A.Villiers det.; Himalaya/ oriental/ R. P. Bertrand // MUSEUM PARIS/ Coll. L. BEDEL 1922; Travancore/ Inde [handwriting] // MUSEUM PARIS/ 1930/ COLL SICARD

#### Distribution.

India.

#### Diagnosis.

The antennal club of this species is only composed of the last four antennomeres, and the outer angle of elytra is more acute in comparison with that of other species in this genus.

### ﻿ Labidolanguria sauteri

Taxon classificationAnimaliaColeopteraErotylidae

(Fowler, 1913) comb. nov.

6DA68D45-7EB8-5974-8282-4C87835CD66C

Fig. 1B

Tetralanguroides sauteri Fowler, 1913: 133. Type locality: China, Taiwan (Taihorin, Kosempo). Type depository: SDEI.
Tetralanguria sauteri : Villiers1945: 290.
Tetraphala sauteri : Leschen and Wegrzynowicz 1998: 241.

#### Type material examined.

***Syntype***: Kosempo/ Formosa/ H. Sauter, 1909 // 7. IX// Fowler det // *Tetralanguroidessauteri* Fowler. / TYPE // Syntypus [red label] // DEI Coleoptera / #200777.

#### Additional material examined.

6 exx. Formosa/ Taihorin / 1911; Kosempo/ Formosa/ 7. IX.1909.

#### Distribution.

China (Taiwan).

#### Diagnosis.

This species can be easily recognized by its antennal club composed of five antennomeres. The outer and sutural angles of the elytra are not very acute.

### ﻿ Labidolanguria apicata

Taxon classificationAnimaliaColeopteraErotylidae

(Zia, 1959) comb. nov.

C8AB0A41-1596-559F-91E1-59CC1C121E92

Fig. 1C

Tetralanguria apicata Zia, 1959: 236. Type locality: China: Sichuan. Type depository: IZCAS.
Tetraphala apicata : Leschen and Wegrzynowicz 1998: 241.

#### Type material examined.

***Holotype***: Sichuan Emei mountain, 1100 m, 1955.VI.22 [In Chinese] / leg. Wu Le [In Chinese] // [in Russian] // HOLOTYPE [red label] // IOZ(E)221361 [blue label] // *Tetralanguriaapicata* m. / det. Zia Yonyon] 1957 [In Chinese]; Paratype: Sichuan Emei mountain, 580–1100 m, 1955.VI.22 [In Chinese] // leg. Huang Tian-rong [In Chinese] // [in Russian] // ALLOTYPE [green label] // IOZ(E)221362 [blue label].

#### Additional material examined.

1♂ 1♀ China: Yunnan Malipo County Xiajinchang Village Zhongzhai / 2015.VI.7 leg. Huang Z.Z. / IZCAS// N 23°07.213′ / E 104°49.378′ / alt. 1855 m / IZCAS.

#### Distribution.

China (Sichuan and Yunnan).

#### Diagnosis.

This species is unique within the genus for its abdominal color pattern; all three ventrites from II to IV have a black or green metallic spot in the middle of the basal margin. The last ventrite has a black or green metallic luster. The abdominal coxal lines are long and parallel.

### ﻿ Labidolanguria liangi

Taxon classificationAnimaliaColeopteraErotylidae

Huang sp. nov.

D0DA8A24-C491-5E7E-8107-46679BC67865

https://zoobank.org/8DDDFDC6-E22B-4332-B3EB-99A6B159DE14

**Figure 2.**
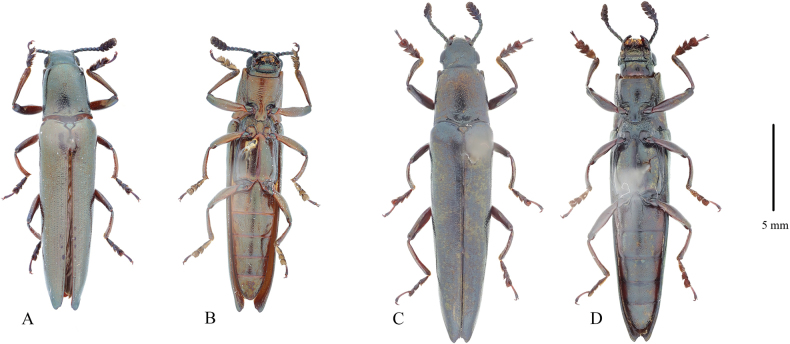
*Labidolangurialiangi* sp. nov. **A, B** holotype, male **A** habitus, dorsal view **B** same, ventral view **C, D** same, paratype, female **C** same, dorsal view **D** same, ventral view. Scale bars: 5.0 mm.

**Figure 3.**
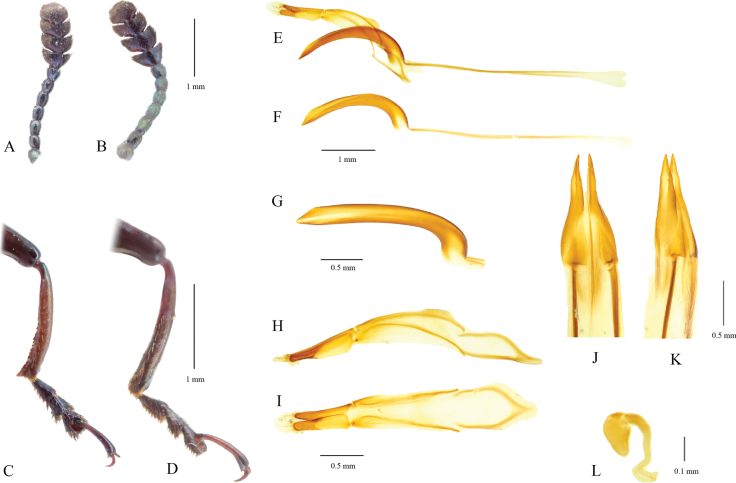
*Labidolangurialiangi* sp. nov. **A** holotype, male antenna **B** paratype, female antenna **C** holotype, male mesotibia **D** paratype, female mesotibia **E–I** holotype, male genitalia **E** male genital, lateral view; **J** median lobe, dorsolateral view **G** apex of median lobe, dorsal view **I** tegmen, dorsal view **H** tegmen, lateral view **J–L** female genitalia, paratype **J** ovipositor, dorsal view **K** same, dorsolateral view **L** spermatheca, lateral view.

Figs 1D , 2A–D , 3A–L

#### Type material.

**China • *Holotype*** male; Xizang Prov., Motuo, CAS; 1980.V.19; Leg. Jin Gen-tao, Wu Jian-yi; 24207168; HOLOTYPE, *Labidolangurialiangi*, det. Huang Z.Z. [red label]. ***Paratypes*. China** • 1♀; Xizang Prov., Motuo Dexing; 970 m; CAS; 1980.VI.1; Leg. Jin Gen-tao, Wu Jian-yi; 24204767 • 1♀; Xizang Prov., Motuo Dexing; 970 m; CAS; 1980.VI.1; Leg. Jin Gen-tao, Wu Jian-yi; 24204769 • 1♀; Xizang Prov., Motuo Kabu; 1200 m; CAS; 1980.V.10; Leg. Jin Gen-tao, Wu Jian-yi; 24203357 • 1♀; Xizang Prov., Motuo Yadong; 1250 m; CAS; 1980.V.25; Leg. Jin Gen-tao, Wu Jian-yi; 24204760 • 1♀; Xizang Prov., Motuo Aniqiao; 1200 m; CAS; 1979.VII.21; Leg. Jin Gen-tao, Wu Jian-yi; 24201228 • 2♀♀; Xizang Prov., Motuo Beibeng; 800–900 m; CAS; 1983.V.15; Leg. Han Yin-heng • 1♀; Xizang Prov., Motuo, Beibeng; 850 m; CAS; 1983.V.15; Leg. Han Yin-heng • 1♂; Xizang Prov., Motuo; 2013.VIII.02–03; Leg. Bai Xing-long, Shan Jun-sheng; Heibei university museum • 1♂; Xizang Prov., Motuo, Beibeng country; 2013.VII.30; Leg. Bai Xing-long, Shan Jun-sheng; Heibei university museum • 1♂; Xizang Prov., Motuo County, Renqinbeng mount; 1314 m; 2015.VIII.26D; IOZCAS; 29.3175°N, 95.3333°E; Leg. Liang Hong-bin, Wang Ming-qiang • 14♀♀, 3♂♂; Xizang Prov., Motuo County, Deguo Bridge; 837 m; 2015.VIII.18; IOZCAS; 29.4019°N, 95.3772°E; Leg. Huang Zheng-zhong • 2♀♀, 1♂; Xizang Prov., Motuo County, Yarang Village; 792 m; 2015.VIII.23; IOZCAS; 29.2964°N, 95.2772°E; Leg. Huang Zheng-zhong • 3♂♂, 2♀♀; Xizang Prov., Motuo County Dexing Country; 796 m; 2015.VIII.25; IOZCAS; 29.3203°N, 95.2911°E; Leg. Huang Zheng-zhong • 3♂♂, 3♀♀; Xizang Prov., Motuo County Motuo Town Lagong Tea plantation; 1253 m; 2021.VI.16D2; IOZCAS; 29.3191°N, 95.3158°E; sweep-net method; Leg. Liang Hong-bin, Xu Yuan et al. • 1♂, 2♀♀; Xizang Prov., Motuo County Motuo Town Lagong Tea plantation; 1294 m; 2021.VI.8D1; IOZCAS; 29.3187°N, 95.3157°E; Leg. Liang Hong-bin, Xu Yuan • 2♂♂; Xizang Prov., Motuo County, Beibeng Country, Gelin Village; 1758 m; 2021.VI.15D1; IOZCAS; 29.2134°N, 95.1717°E; Leg. Liang Hong-bin, Xu Yuan • 1♂; Xizang Prov., Motuo County, Beibeng Country, Jiangxin Road 2 km; 830 m; 2021.VI.12N1; IOZCAS; 29.2325°N, 95.1461°E; light trap method; Leg. Liang Hong-bin et al. • 1♀; Xizang Prov., Linzhi City, Motuo County, Beibeng Country, Jiaga Valley; 677 m; 2021.VI.13; IOZCAS; 29.2540°N, 95.1965°E; Leg. Liu Hong • 1♀; Xizang Prov., Motuo County, Beibeng Country, Jiangxin Village; 764 m; 2021.VI.13; IOZCAS; 29.2240°N, 95.1311°E; Leg. Liu Hong.

#### Distribution.

Known only from Motuo County (China, Xizang Autonomous Region).

#### Diagnosis.

This species resembles *L.mucronata* in body structures, but it is distinguished by its darker, duller coloration, and the apex of the elytra which is not as acute. Moreover, *L.liangi* exhibits sexual dimorphism at the base of the mesotibia: the male mesotibial segment terminates medially with some denticulations and a prominent terminal tooth, while the female lacks these features.

#### Description.

Body length 12.1–17.1 mm, width 2.4–3.3 mm. Body with coppery, metallic luster. Ventral side dark or black with metallic luster. Abdomen deep or pale brown; middle of ventrites I–IV nearly black and with deep green metallic luster; last ventrite always black and with metallic luster. Coxae and trochanters red-brown; femora and tibiae with green metallic luster. Tarsomeres I–III with indigo metallic luster. Antennomeres I–VII with green metallic luster; antennomeres VIII–XI with indigo metallic luster (Fig. 3A, B).

Body slender and subparallel. Head with dense punctures, coarsest near eyes but finer between eyes. Antennae 11 antennomeres; club pubescence composed of four antennomeres. Compound eyes medium-sized, finely faceted. Clypeofrontal suture obvious; clypeus wider than long, with dense punctures. Anterior edge of clypeus straight or sometimes concave in middle.

Pronotum slightly convex, nearly rectangular, longer than broad, finely punctate; sides subparallel, but middle part of lateral pronotal carina invisible from above. Anterior angle round, thick, and not produced; posterior angle not acute but produced, reaching elytral humeri. Basal fovea deep, with one pair of short, deep lateral fovea. Basal margin complete and clear.

Prosternum coarsely punctate and plicated, without setae. Prosternal process long and trapezoidal, weakly convex in middle, with fine punctures. Each lateral side with deep groove; apex of prosternal process broad.

Scutellar shield liguliform, with round apex. Elytra parallel before apex, regularly striate-punctate, and with intervals with fine punctation. Apices of elytra tapering and acute; sutural angle distinctly acute and produced; outer angle of elytra not acute as sutural angle but more produced.

Mesoventrite coarsely and densely punctate. Median suture of metaventrite not reaching apex of metaventral process. Abdomen finely punctate, with one pair of long, parallel coxal lines, reaching half of ventrite 1. Last ventrite with dense, yellow setae at apex. Male genitalia similar to those congeners in the genus. Median lobe long and slender, slightly curved. Apex of median lobe somewhat acute (Fig. 3E–G); internal sac invisible. Paramere slender, with apex not produced and with short yellow setae (Fig. 3H, I). Ovipositor short, with apex sclerotized and acute, without stylus and setae (Fig. 3J, K). Spermathecal capsule heart-shaped (Fig. 3L).

#### Dimorphism.

The sexual dimorphism of this species is mainly reflected in the varying number of spines along the inner side of the mesotibia in males, with sometimes large, prominent terminal spines (Fig. 3C, D). This feature is not found in other species of the genus and is very rare even at the subfamily Languriinae in general. Some languriines exhibit sexual dimorphism in the form of protuberances on the profemur of males, such as in the genera *Doubledaya* and *Callilanguria* (Huang et al. 2018).

#### Etymology.

The specific epithet honors Dr. Hong-bin Liang, a specialist of Carabidae from the Institute of Zoology, Chinese Academy of Sciences, for his outstanding leadership and contribution during multiple expeditions in Yunnan and Xizang provinces.

#### Host plant.

**Figure 4.**
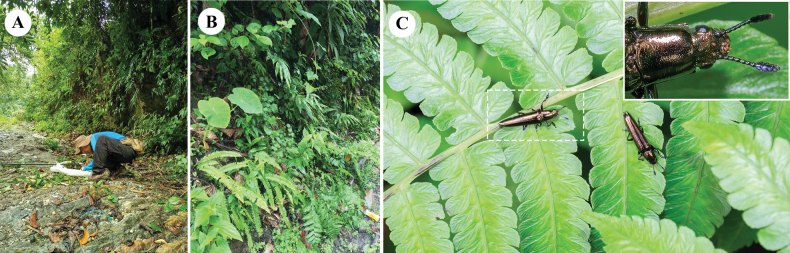
**A, B** the environment at the time of collecting, in Motuo County **C***Labidolangurialiangi* sp. nov. feeding on spores on surface of fern leaf, and (inset) spores on beetle mouthparts. Photographs by Dr. Zheng-Zhong Huang.

In the field, we observed that this new species is relatively common in shrublands mixed with plants outside Motuo County and along the Renqingbeng Mountain Road (Fig. 4A, B). It lives on *Diplazium* sp. (family Athyriaceae) and was observed feeding on spores of this fern (Fig. 4C).

#### Variation.

The variation of this species is mainly reflected in its body color and the spines on the mesotibia of males. Early collected specimens generally have a brownish body with a weak metallic luster. However, the specimens we collected in Motuo in recent years have a relatively dark -green body with a slight metallic luster. The differences in the spines on the inner side of the mesotibia of males among different individuals are also large, some are obvious, while others are not very prominent.

## ﻿Discussion

Compared with the fungivorous erotylids, herbivorous languriines are rare in the family, yet their hosts remain largely unknown. Toki et al. (2020) recorded and summarized the hosts of some known species of adult and larval languriines. Some species of *Tetraphala* feed on ferns, and consistent with our unpublished data, the species of ferns they feed on belong to different families or genera.

Fowler (1908) established this genus and considered it similar to *Pentelanguria*, and both morphological characteristics and feeding habits indeed suggest that *Labidolanguria* and *Tetraphala* are rather closely related. The only difference between them is in shape of the elytral apex, which is why these two new combinations of *Labidolanguria* were initially assigned to *Tetralanguria*. (*Tetralanguria* was treated as a synonym of *Tetraphala* by Leschen and Wegrzynowicz 1998). In terms of feeding habits, both genera include relatively rare insects that feed on ferns. According to the latest review of phytophagous Coleoptera (Fuentes-Jacques et al. 2021), groups that feed on ferns account for 0.5% of currently recorded species with known feeding habits. *Labidolanguria* is here treated as a genus separate from *Tetraphala*, although it might be better to regard it as a subgenus. Further research on the phylogenetic relationships within Languriinae is essential to clarify this problem.

## Supplementary Material

XML Treatment for Labidolanguria

XML Treatment for Labidolanguria mucronata

XML Treatment for Labidolanguria sauteri

XML Treatment for Labidolanguria apicata

XML Treatment for Labidolanguria liangi

## ﻿Additional information

### Author ORCIDs

Zheng-Zhong Huang https://orcid.org/0000-0002-0085-6218

Xing-Ke Yang https://orcid.org/0000-0002-0870-4657

Si-Qin Ge https://orcid.org/0000-0001-5924-3400

### Data availability

All of the data that support the findings of this study are available in the main text.
